# Does Protocatechuic Acid Affect the Activity of Commonly Used Antibiotics and Antifungals?

**DOI:** 10.3390/life12071010

**Published:** 2022-07-07

**Authors:** Adrian Fifere, Ioana-Andreea Turin-Moleavin, Irina Rosca

**Affiliations:** Centre of Advanced Research in Bionanoconjugates and Biopolymers, “Petru Poni” Institute of Macromolecular Chemistry, 700487 Iasi, Romania; fifere@icmpp.ro (A.F.); moleavin.ioana@icmpp.ro (I.-A.T.-M.)

**Keywords:** protocatechuic acid, drug resistance, antibiotics, antifungals, increased activity

## Abstract

The aim of this study is to evaluate the efficiency of protocatechuic acid (PCA) in enhancing the commonly used drugs used to fight against nosocomial infection. These drugs are represented by routinely used antibiotics, synthetic chemotherapeutic agents with an antimicrobial spectrum, and antifungals. Three concentrations of PCA were added to 12 types of commercial disks used for antibiotic and antifungal susceptibility and tested against bacterial and yeast strains represented by *Staphylococcus aureus*, *Escherichia coli*, *Pseudomonas aeruginosa*, and *Candida albicans*. The results proved that PCA increased up to 50% of the antibacterial activity, especially that of levofloxacin against *Staphylococcus aureus* and *Escherichia coli*. These formulations will lead to new drug design ideas containing a smaller amount of antibiotics with the same effectiveness.

## 1. Introduction

Bacterial infections are a major challenge in modern medicine due to the well-known fact that many micro-organisms become resistant to antibiotics and antifungals over time [1], resulting in approximately 700,000 deaths each year [2]. As a result, drug resistance is becoming an Achilles heel in modern medicine [3].

However, despite reports of clinical failure of antibiotics and antifungals with in vitro resistance detection, they are still in use today [4]. One of the implications is that patients are at risk of developing multidrug resistance [5]. Even the broad-spectrum cephalosporins of the third generation, such as ceftazidime and cefotaxime, should be used sparingly due to their reported drug resistance [6]. Apart from that, in clinical practice, antibiotics of beta-lactam class, including amoxicillin, cephalosporins, and carbapenems, used to treat Gram-negative bacterial infections, are not recommended to be administered alone due to the difficulty of resistance mechanisms (a metallo-lactamase enzyme produced by Gram-negative bacteria can inhibit beta-lactam antibiotics) [7].

Recently, the group of Prof. Andersson identified resistance against nitrofurantoin [3]. Nitrofurantoin is a synthetic derivative of nitrofuran antibiotics that inhibits DNA, RNA, and cell wall protein synthesis and is used against urinary tract infections [8,9,10].

Due to the extensive use of trimethoprim antibiotics [11,12] in the treatment of urinary tract infections, antimicrobial resistance has increased, pushing researchers to pursue a deeper understanding of the mechanisms of resistance in order to improve the drug’s future use [13]. A major source of morbidity in hospitals, nosocomial infections are caused by catheter-associated urinary infections, which are resistant to ampicillin (40–100%), cotrimoxazole (75–90%), tetracycline (66.7–86.6%), and ciprofloxacin (69%), as well as up to 71.4% resistant to norfloxacin [14]. Drug resistance has also emerged in the treatment of respiratory infections. In a recent study, the group of Sediana showed that in the treatment of respiratory infections with levofloxacin, 50% of 142 patients had high levels of resistance, 30.95% had intermediate levels of resistance, and 19.04% of patients were still sensitive to the antibiotic [15].

Besides antibiotics, antifungal drugs are also used to treat various mycoses. These can be polyene antifungal drugs (amphotericin B and nystatin), which bind to ergosterol, a component of the cell membrane of fungi, or azole antifungal drugs (ketoconazole, econazole, and fluconazole), which inhibit the conversion of lanosterol to ergosterol [16]. It is worth noting that amphotericin B, is the “gold-standard” antifungal for *Candida* infections and has been used in clinical practice for more than 40 years [17]. However, growing drug resistance and the negative side effects of antifungal medications limit their clinical uses [18,19,20]. Finally, expanding the usage of medicine requires its efficacy to be increased and its toxicity to be reduced.

In this regard, new approaches are needed to achieve effective therapy despite resistance, as well as to reduce the emergence of resistance, long-term persistence, or collateral sensitivity, which can increase the susceptibility of drugs by a factor of two to four [3].

To eliminate or reduce drug resistance, it is important to lower doses by using synergistic interactions with other compounds, such as natural antioxidants extracted from plants [21]. One such compound is protocatechuic acid (PCA), a phenolic compound found in medicinal plants, spices, and traditional Chinese herbal medicines exhibiting many important activities, such as antioxidant, antibacterial, and antiviral activities [22]. PCA is a widely distributed bioactive compound that it is found in many plants, such as *Euterpe oleracea*, *Allium cepa*, *Daucus carota*; mushrooms, such as *Agaricus bisporus* and *Phellinus linteus*; loquat fruit; honey; soybean; fruits of *Ficus* species; spices, such as *Illicium verum*, *Melissa officinalis*, *Rosmarinus officinalis*, and *Cinnamomum aromaticum* [23]; or medicinal plants, such as *Ginkgo biloba*, *Cibotium barometz*, and *Stenoloma chusanum* [22].

The PCA antioxidant action comes from the property of scavenging free radicals by donating hydrogen atoms or electrons [24], whereas the antibacterial activity is due to the action of decreasing lipid oxidation levels [25], and its antiviral property is due to the replication of the hepatitis B virus [26]. Our group also loaded unstable and insoluble PCA into magnetic cargo complexes under mild conditions using a simple and reproducible approach to increase its bioavailability and its continuous pharmaceutical effects, by releasing it for more than three days [27,28,29,30].

Within this context, the aim of this research is to test the synergistic effect of PCA and commonly used antibiotics/synthetic chemotherapeutic agents with antimicrobial spectra (such as amoxicillin/clavulanic acid, ceftazidime, gentamicin, nitrofurantoin, and cefotaxime) and antifungals (such as amphotericin B, nystatin, econazole, ketoconazole, cotrimoxazole, and fluconazole) against four different reference strains (*Staphylococcus aureus* ATCC25923, *Escherichia coli* ATCC25922, *Pseudomonas aeruginosa* ATCC27583, and *Candida albicans* ATCC10231) to fight nosocomial infections, as well as to aid in the development of drug switches to combat drug resistance.

## 2. Materials and Methods

### 2.1. Materials

PCA (3,4-Dihydroxybenzoic acid) was purchased from Sigma Aldrich (Merck, Darmstadt, Germany). PCA solutions (50 µg/mL PCA_1_, 100 µg/mL PCA_2_, and 200 µg/mL PCA_3_) were prepared in DMSO. Amoxicillin/clavulanic acid, 20/10 µg/disc (AMC); ceftazidime, 30 µg/disc (CAZ); gentamicin, 10 µg/disc (GEN). Levofloxacin, 5 µg/disc (LEV); nitrofurantoin, 300 µg/disc (NIT), cotrimoxazole, 25 µg/disc (COT) from Tody Laboratories (Bucharest, Romania); and cefotaxime, 100 µg/disc (CTX) from HiMedia Laboratories (Mumbai, India). Amphotericin B, 100 µg/disc (AP) from HiMedia Laboratories (Mumbai, India); nystatin, 100 µg/disc (NS); econazole, 10 µg/disc (ECO); ketoconazole, 10 µg/disc (KT) from Tody Laboratories (Bucharest, Romania); and fluconazole, 10 µg/disc (FLC) from Bioanalyse Limited (Ankara, Turkey).

### 2.2. Methods

The antimicrobial activity of the samples (commercial discs, PCA solutions, and their combinations) was determined by disk diffusion assay [31,32] against four different reference strains (*Staphylococcus aureus* ATCC25923, *Escherichia coli* ATCC25922, *Pseudomonas aeruginosa* ATCC27583, and *Candida albicans* ATCC10231) according to the recommendations of the disc manufacturer. All micro-organisms were stored at −80 °C in 20% glycerol. The bacterial strains were refreshed on Mueller-Hinton agar at 37 °C, and the fungal strain was refreshed on Sabouraud dextrose agar at 37 °C. Microbial suspensions were prepared with these cultures in sterile solution to obtain turbidity optically comparable to that of 0.5 McFarland standards. Volumes of 0.2 mL from each inoculum were spread onto Mueller–Hinton agar and Sabouraud dextrose agar. The sterilized paper discs were placed on the inoculated Petri plates. Aliquots of 15 μL of the tested PCA concentrations were added to the paper discs. To evaluate the antimicrobial properties, growth inhibition was measured under standard conditions after 24 h of incubation at 37 °C. All tests were carried out in triplicate to verify the results. After incubation, the diameters of inhibition zones were measured using Image J software [33]. The ratio of amplification of antibacterial/antifungal activity is defined as: % = (*Izb* − *Iza*)/*Iha* × 100%, where *Iza* and *Izb* are the average values (mm) of inhibition zone of the control drug and drug-PCA, respectively.

### 2.3. Statistical Analysis

Results of antibacterial activity are expressed as the mean ± standard deviation (SD) and were subjected to one-way analysis of variance (ANOVA), with *p* < 0.05 considered statistically significant, using XLSTAT Ecology software [34].

## 3. Results

**Figure 1:**
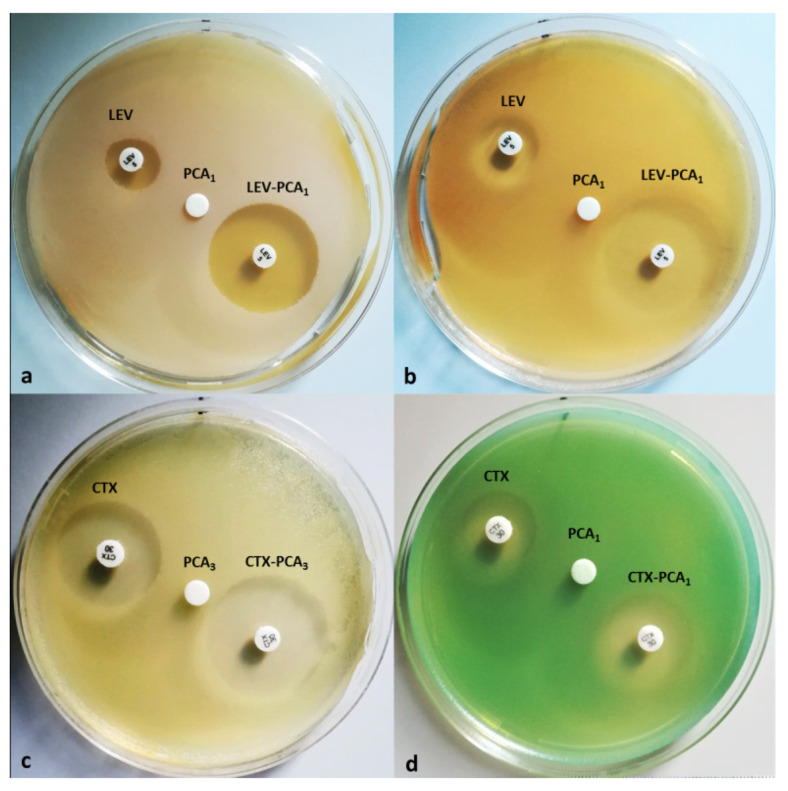
PCA enhances the activity of levofloxacin (LEV) against *Staphylococcus aureus* (**a**) and *Escherichia coli* (**b**), as well as that of cefotaxime (CTX) against *Escherichia coli* (**c**) and *Pseudomonas aeruginosa* (**d**).

In the present study, we examined the evolution of the inhibition of four reference strains (*Staphylococcus aureus* ATCC25923, *Escherichia coli* ATCC25922, *Pseudomonas aeruginosa* ATCC 27583, and *Candida albicans* ATCC10231) when PCA formulations were administered with common antibiotics or other classes of antimicrobials (Figure 1, Table 1 and Table 2).

Screening included drugs (antibiotics/synthetic chemotherapeutic agents with antimicrobial spectra/antifungals) from the classes of penicillin beta-lactams (amoxicillin/clavulanic acid (AMC)), fluoroquinolones (levofloxacin (LEV)); cephalosporins (ceftazidime (CAZ) and cefotaxime (CTX)), nitrofurans (nitrofurantoin (NIT), aminoglycosides, and gentamicin (GEN)); polyenes (amphotericin B (AP) and nystatin (NS)), imidazole (econazole (ECO), ketoconazole (KT), cotrimoxazole (COT)), and triazole (fluconazole (FLC)). The investigated strains were treated with combinations of PCA at three different concentrations (PCA_1_, 50 µg/mL; PCA_2_, 100 µg/mL; and PCA_3_, 200 µg/mL) with drugs from different classes, as shown in Figure 1 and Table 1: (i) *Candida albicans* was treated with combinations of PCA_1÷3_ and several antifungals of different classes (AP, NS, ECO, KT, and FLC); (ii) *Staphylococcus aureus* and *Escherichia coli* were treated with combinations of PCA_1÷3_ and seven dedicated antibiotics (AMC, LEV, CAZ, CTX, GEN, NIT, and COT); and (iii) *Pseudomonas aeruginosa* was treated with combinations of PCA_1÷3_ and four specific drugs (CAZ, CTX, GEN, and LEV).

Careful examination of the results (Figure 1 and Table 1 and Table 2) shows that increased antimicrobial activity of some drugs was obtained when the concentration of PCA was 50 µg/mL, whereas higher PCA concentration had no effect, with one exception in the case of CTX, for which PCA_3_ (200 µg/mL) increased the activity by 20.30% against *Escherichia coli*. It is also worth noting that adding PCA_1_ to LEV had a synergistic impact, boosting LEV and GEN antibacterial activities by 55.98% and 20.55%, respectively, against *Staphylococcus aureus*. Additionally, PCA_1_ improved the activity of LEV (28.45%), NIT (26.13%), and COT (29.66%) against the Gram-negative strain represented by *Escherichia coli*. PCA_1_ also improved the antibacterial activity of CTX and LEV by 13.59% and 15.25%, respectively, against *Pseudomonas aeruginosa*. Analysis of variance revealed that the mean bacterial growth inhibition zone was significantly influenced by the tested PCA–drug combinations (*Staphylococcus aureus*, *p* ˂ 0.009; *Escherichia coli*, *p* ˂ 0.007; *Pseudomonas aeruginosa*, *p* ˂ 0.050).

## 4. Discussion

Over the last decade, with respect to drug resistance, the research studies have considered the synergic effect produced by phytochemicals on antifungal medications [35]. One of these phytochemicals is PCA, found in medicinal plants, spices, and traditional Chinese herbal medicines in different concentrations. For example, in *Ginkgo biloba*, the content of free phenolic acids (protocatechuic and p-hydroxybenzoic acids) is 19.69 µg/g in fresh leaves and 345.32 µg/g in roots, where PCA is predominant [22]. *Cibotium baromez*, a traditional Chinese herbal medicine, presents a content of total phenolic acid of 3.73–6.16% in rhizome [36], whereas *Hibiscus sabdariffa*, with a well-known antiseptic effect, has a PCA content of 8.62% [37]. *Allium cepa*, which is recognized for its antifungal properties [22], presents a total flavonoids content (TFC) of 55.27 mg, with a total polyphenolic content (TPC) of 97.28 mg [38]. A product that is rich in PCA is represented by Acai palm (*Euterpe oleracea*), the oil of which has a 630 mg/kg PCA content [21]. PCA is also found in large quantities in various fruits and spices, such as: blackberries, 127 μmol/kg; mulberry, 119 μmol/kg; raspberry, 270 μmol/kg; strawberry, 112 μmol/kg; cranberry; gooseberry, 405 μmol/kg; dried dates, 320 μmol/kg; and star anise, 2.090 μmol/kg [39].

PCA is known in the literature to produce antibacterial effects due to its ability to inhibit bacterial growth. Inhibitory mechanisms of phenolic acids with respect to bacterial growth are nonspecific that can involve various modes of action, such as destabilization and permeabilization of the bacterial cytoplasmatic membrane; inhibition of virulence factors, such as enzymes and toxins, directly altering microbial metabolism; and inhibition of bacterial biofilm formation [40,41,42]. The main factor leading to these inhibitory actions is considered to be the hydroxyl groups of phenolic compounds, which can interact with the cell membrane of bacteria and damage the structures of the cell membrane [43]. Plant phenolic compounds have been considered powerful antibacterial agents, particularly for preventing the formation of biofilms by producing changes in the bacterial surface, reduction in exopolysaccharide production, and interference with cell communication [40,44,45]. Additionally, certain phenolic compounds function by a different mechanism than the known antibacterial agent. Different combinations may enhance or facilitate an antibiotic’s engagement with its target inside the bacterial cell. Because lower dosages of both medicines can be utilized, the synergy can be used to broaden the antibacterial range, prevent the emergence of micro-organisms resistant to antibiotics, and minimize toxicity [45]. Furthermore, it has been reported that phenolic acids might damage the cytoplasmic membrane’s structure, resulting in a loss of integrity and eventual cell death [46]. Lower antibiotic doses would be required because the compounds in the extract, at sub-inhibitory concentrations, would facilitate the antibiotic’s entry into the cell cytoplasm, allowing fluoroquinolones, tetracycline, and chloramphenicol, which have their sites of action inside the bacterial cell, to enter more easily [47].

In this regard, several cases of phytochemicals improving the activity of the antibiotics have been presented in the literature. Jayaram et al. [48] studied the activity and interactions of antibiotics and phytochemicals against *Pseudomonas aeruginosa* in vitro. They tested seven different antibiotics (ciprofloxacin, tetracycline, ceftazidime, sulfamethoxazole, polymyxin B, trimethoprim, and piperacillin) in combination with six phytochemicals (protocatechuic acid, ellagic acid, gallic acid, berberine, rutin, and myricetin), and the results indicated that pairs of active principles, such as PCA–sulfamethoxazole, ellagic acid–sulfamethoxazole, and gallic acid–sulfamethoxazole have synergistic effects against *Pseudomonas aeruginosa*. In our study, the most promising result was achieved in the case of CTX and LEV in combination with PCA_1_, with their activities were increased by 13.59% and 15.25%, respectively, against the same bacterial strain. 

Another study on antibiotic–phytochemical effects, was carried out by Kyaw, BM et al. [49], in which fusidic acid, rifampicin, cefotaxime, vancomycin, and ofloxacin were used in combination with six phytochemicals (tannic acid, quercetin, gallic acid, caffeic acid, eugenol, and menthone) against *Staphylococcus aureus*. The results indicated that tannic acid was synergistic with cefotaxime, rifampicin, fusidic acid, and minocycline, whereas quercetin was synergistic with fusidic acid, minocycline, and rifampicin. In this regard, our results show that the combinations of PCA_1_ and LEV or GEN drugs exhibited a 55.98% and 20.55%, boost in activity against the same strains, respectively.

This is a preliminary study demonstrating that simple combinations of well-studied substances, such as regularly used antibiotics and PCA, can be more effective than the pure antibiotic. We aim to use the promising drug–PCA combinations to generate nanoformulations consisting of magnetically driven therapeutic agents with the goal of lowering antibiotic dosages while retaining efficacy. These findings will have a positive impact on the development of novel pharmaceutical formulations as weapons in the fight against antibiotic resistance in bacteria.

## 5. Conclusions

This in vitro study proves that PCA addition can significantly improve the antibacterial activity of commonly used drugs, such as levofloxacin, nitrofurantoin, and cotrimoxazole, against both Gram-positive and Gram-negative bacterial strains by up to 50%.

This is the first report on the possible beneficial effects of PCA in the administration of the drugs presented herein. Considering the results presented in this brief report, further studies are needed to understand the detailed aspects of the extent and the manner in which PCA and antibiotics/synthetic chemotherapeutic agents with a broad antimicrobial spectrum influence each other. However, these formulations will lead to new drug design ideas that contain a smaller amount of antibiotics with the same effectiveness.

## Figures and Tables

**Table 1:** Antibacterial activity of the tested compounds against the reference strains.

Tested Drugs	*Staphylococcus aureus*		*Escherichia coli*		*Pseudomonas aeruginosa*	
	Inhibition Zone	% *	Inhibition Zone	% *	Inhibition Zone	% *
AMC	37.75 ± 0.56	-	22.06 ± 0.71	-	-	-
CAZ	16.01 ± 0.46	-	29.82 ± 0.74	-	31.56 ± 0.85	-
CTX	29.04 ± 0.17	-	28.13 ± 0.59	-	19.26 ± 0.61	-
GEN	19.62 ± 0.28	-	19.49 ± 0.85	-	17.40 ± 0.63	-
LEV	20.66 ± 0.91	-	29.84 ± 0.13	-	23.48 ± 0.84	-
NIT	19.96 ± 0.09	-	22.86 ± 0.01	-	-	-
COT	23.73 ± 0.21	-	23.05 ± 0.60	-	-	-
PCA_1_	0	0	0	0	0	0
PCA_2_	0	0	0	0	0	0
PCA_3_	0	0	0	0	0	0
AMC-PCA_1_	39.85 ± 0.05	5.57	22.07 ± 0.01	0.10	-	-
AMC-PCA_2_	40.74 ± 0.12	7.93	22.46 ± 0.07	1.79	-	-
AMC-PCA_3_	43.16 ± 0.52	14.33	22.80 ± 0.04	3.33	-	-
CAZ-PCA_1_	14.97 ± 0.13	−6.48	28.03 ± 0.08	−6.00	30.36 ± 0.03	−3.80
CAZ-PCA_2_	16.15 ± 0.05	0.89	30.22 ± 0.23	1.34	29.77 ± 0.29	−5.67
CAZ-PCA_3_	14.98 ± 0.15	−6.43	29.76 ± 0.12	−0.25	32.85 ± 0.26	4.09
CTX-PCA_1_	31.31 ± 0.05	7.78	28.99 ± 0.35	3.04	21.88 ± 0.55	13.59
CTX-PCA_2_	30.45 ± 0.12	4.84	28.88 ± 0.25	2.66	19.35 ± 0.15	0.48
CTX-PCA_3_	29.55 ± 0.02	5.36	33.85 ± 0.32	20.30	19.04 ± 0.02	−1.34
GEN-PCA_1_	35.02 ± 0.01	20.55	19.26 ± 0.08	−1.14	19.21 ± 0.10	10.49
GEN-PCA_2_	23.18 ± 0.02	−20.18	19.76 ± 0.05	1.39	16.56 ± 0.02	−4.84
GEN-PCA_3_	24.92 ± 0.05	−14.20	19.47 ± 0.12	−0.09	17.81 ± 0.08	2.34
LEV-PCA_1_	32.24 ± 0.01	55.98	38.34 ± 0.37	28.45	28.43 ± 0.03	15.25
LEV-PCA_2_	35.79 ± 0.04	43.15	32.68 ± 0.28	10.11	25.02 ± 0.04	4.95
LEV-PCA_3_	34.84 ± 0.45	38.60	29.67 ± 0.50	−0.56	26.38 ± 0.10	10.65
NIT-PCA_1_	18.10 ± 0.22	−9.29	28.84 ± 0.26	26.13	-	-
NIT-PCA_2_	19.86 ± 0.04	−0.47	24.15 ± 0.08	5.61	-	-
NIT-PCA_3_	19.57 ± 0.02	−1.93	24.33 ± 0.20	6.40	-	-
COT-PCA_1_	24.92 ± 0.12	5.01	29.87 ± 0.08	29.66	-	-
COT-PCA_2_	24.24 ± 0.03	2.13	23.85 ± 0.07	3.45	-	-
COT-PCA_3_	24.64 ± 0.58	3.83	23.81 ± 0.29	3.27	-	-

PCA, protocatechuic acid; AMC, amoxicillin/clavulanic; CAZ, ceftazidime; GEN, gentamicin; LEV, levofloxacin; NIT, nitrofurantoin; COT, cotrimoxazole; CTX, cefotaxime; PCA_1_, 50 µg/mL; PCA_2_, 100 µg/mL; PCA_3_, 200 µg/mL; * percentage from initial drug activity; -, not tested. Data are represented as mean ± standard deviation of experiments performed in triplicate.

**Table 2:** Antifungal activity of the tested compounds against *Candida albicans*.

Tested Drug	*Candida albicans*	
	Inhibition Zone	% *
AP	20.59 ± 0.37	-
NS	23.22 ± 0.29	-
ECO	21.30 ± 0.68	-
KT	18.46 ± 0.29	-
FLC	28.07 ± 0.69	-
PCA_1_	0	0
PCA_2_	0	0
PCA_3_	0	0
AP-PCA_1_	16.18 ± 0.08	−2.28
AP-PCA_2_	20.16 ± 0.05	−1.91
AP-PCA_3_	20.86 ± 0.12	1.49
NS-PCA_1_	24.77 ± 0.45	6.66
NS-PCA_2_	24.03 ± 0.25	3.45
NS-PCA_3_	21.22 ± 0.18	−8.62
ECO-PCA_1_	19.05 ± 0.07	−1.52
ECO-PCA_2_	19.61 ± 0.12	−7.93
ECO-PCA_3_	19.67 ± 0.35	−7.66
KT-PCA_1_	17.76 ± 0.07	−3.81
KT-PCA_2_	18.75 ± 0.27	1.58
KT-PCA_3_	19.23 ± 0.14	4.18
FLC-PCA_1_	26.91 ± 0.12	−4.15
FLC-PCA_2_	26.08 ± 0.11	−7.36
FLC-PCA_3_	26.05 ± 0.07	−7.37

AP, amphotericin B; NS, nystatin; ECO, econazole; KT, ketoconazole; FLC, fluconazole; PCA_1_, 50 µg/mL; PCA_2_, 100 µg/mL; PCA_3_, 200 µg/mL; * percentage from initial drug activity; -, not tested. Data are represented as mean ± standard deviation of experiments performed in triplicate.
